# Short Mindfulness Meditations During Breaks and After Work in Everyday Nursing Care: A Simple Strategy for Promoting Daily Recovery, Mood, and Attention?

**DOI:** 10.1177/21650799241262814

**Published:** 2024-08-28

**Authors:** Elisabeth M. Riedl, Johanna Perzl, Kathrin Wimmer, Janusz Surzykiewicz, Joachim Thomas

**Affiliations:** 1 Department of Psychological Assessment and Intervention, Catholic University of Eichstätt-Ingolstadt; 2 Department of Work and Organizational Psychology, Julius Maximilian University of Würzburg; 3 Urban Yoga Walks; 4 Chair of Social and Health Pedagogy, Catholic University of Eichstätt-Ingolstadt; 5 Chair of Psychological Foundations of Pedagogy, Cardinal Stefan Wyszynski University

**Keywords:** mindfulness/meditation/yoga, ecological momentary intervention, break recovery, psychological detachment, ambulatory attention measurement, nursing, employee health, health promotion

## Abstract

**Background:**

Nurses experience high job demands, which makes recovery particularly necessary to maintain well-being and performance. However, these demands also make recovery challenging. Short mindfulness meditations could potentially help alleviate this paradox.

**Methods:**

Two ecological momentary intervention studies were conducted among geriatric nurses (Study 1: break study) and hospital nurses (Study 2: after-work study) to investigate whether short audio-guided mindfulness meditations are beneficial for recovery during breaks and psychological detachment after work. Furthermore, break recovery and after-work detachment were examined as mediators of the associations between mindfulness meditations and after-break/after-sleep mood and attention after respective recovery periods. Multilevel path models were based on a sample of 38 nurses and 208 after-break surveys in the break study and 26 nurses and 192 after-sleep surveys in the after-work study.

**Results:**

Compared to breaks spent as usual, breaks that incorporated short mindfulness meditations were associated with higher break recovery, which mediated the positive associations between mindful breaks and after-break calmness, valence, and energetic arousal. Only with certain constraints did mindfulness meditations predict a lower rate of attention failures. In the after-work study, short mindfulness meditations were positively related to psychological detachment, which mediated the positive associations between the intervention and after-sleep valence and calmness.

**Conclusion/Application to Practice:**

Both pilot studies showed that short mindfulness meditations aid in recovery among nurses. However, to fully utilize the advantages of recovery-promoting breaks, structural changes are necessary to ensure that breaks of an appropriate duration are consistently implemented.

## Background

When confronted with high work demands, rest is crucial for maintaining well-being and the ability to work (Sonnentag, 2018). On the other hand, recovery is difficult, especially when demands are high—a phenomenon known as the recovery paradox (Sonnentag, 2018). One type of occupation that is likely to be particularly affected by this paradox is nursing, where an extraordinarily high level of work intensity is coupled with high physical and emotional demands and unfavorable working time conditions (Bundesanstalt für Arbeitsschutz und Arbeitsmedizin, 2020). In light of this stressful working environment, recovery is decisive for maintaining physical and mental health (Kellmann et al., 2023). Recovery is defined as the process that reverses load reactions and restores the resource pool (Sonnentag & Fritz, 2007) and can be exercised during different periods, for example, vacations, free time between working days, and rest breaks (Sonnentag et al., 2017). Break recovery is very problematic in nursing, as breaks are much too short, delayed or do not take place at all, or are interrupted (Wendsche et al., 2022). Regarding recovery after a shift, nurses report a higher need for recovery than other occupations (Moriguchi et al., 2012; Sluiter et al., 2003), which manifests in the perceived urgency to recreate resources but simultaneously lacking power to engage in recreational activities (Sonnentag & Fritz, 2007).

## Aim and Contribution of the Two Studies

Short mindfulness meditations could be a feasible and effective way to improve recovery in nursing. Mindfulness interventions aim at increasing state mindfulness (Creswell, 2017), which can be defined as “being attentive to and aware of what is taking place in the present” (Brown & Ryan, 2003, p. 822). Via associations with cognition, emotion, behavior, and physiology, this attentional state can be connected to well-being, social relationships, and performance at work (Good et al., 2016). While some authors propose that mindfulness meditations have an enormous potential in nursing (Green & Kinchen, 2021), there are studies indicating that mindfulness interventions may not be feasible in such high-stress contexts (Moody et al., 2013). Steed et al. (2021) showed in their meta-analysis that work demands are basically not related to the use of low-duty recovery activities such as meditation. Among nurses, both break intention and control are positively associated with break behavior (Blasche et al., 2021). Both of these aspects—intention and control—may be strengthened by guided recovery activities. However, it is unclear whether mindfulness meditations are actually feasible in stressful day-to-day care routines, which is therefore the first concern of this study.

**Figure 1.**
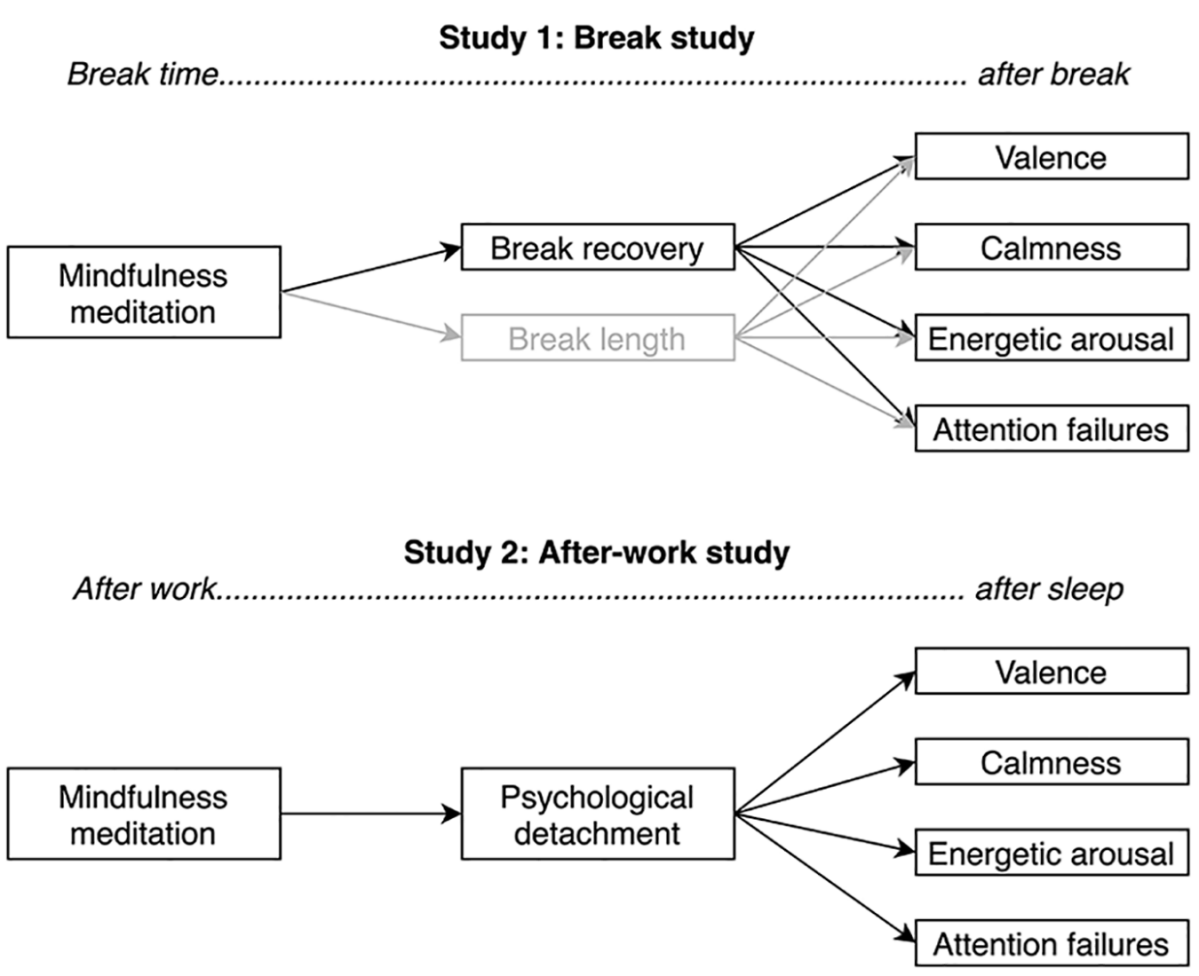
Graphical Illustration of the Proposed Within-Person Path Model of the Break Study (Study 1) and the After-Work Study (Study 2)

Due to the high time expenditure of traditional programs, there is a growing interest in short mindfulness interventions (Gilmartin et al., 2017). Rodriguez-Vega et al. (2020), for example, recently showed that 5–10 minutes of in-person guided mindfulness exercises in a hospital setting during the COVID-19 crisis were perceived as helpful by hospital staff for reducing stress. We propose to expand this concept of short mindfulness meditations in everyday life. As in-person interventions consume considerable time and monetary resources, mobile-based interventions may represent an interesting alternative (Egger et al., 2023). Thus, we conducted two pilot studies to investigate whether short audio-guided mindfulness meditations during breaks and after shifts present a low-threshold, feasible, and effective measure to promote break recovery and after-work psychological detachment in a sample of geriatric nurses (Study 1: break study) and hospital nurses (Study 2: after-work study). Furthermore, break recovery and after-work psychological detachment were examined as mechanisms that may mediate time-lagged relationships between meditation and after-break and after-sleep mood. However, successful recovery is related to both the affective state and also the restoration of cognitive resources. Thus, in addition to self-reported mood, this study also assessed objectively measured attention performance, which had been requested repeatedly (Kim et al., 2017; Sonnentag et al., 2017). Due to the connection between cognitive failures and patient safety (Park & Kim, 2013), reduced attention due to insufficient recovery is of great practical relevance. Figure 1 illustrates the proposed research models. The relevance of the variable break length is described below and in the Methods section.

We applied the method of ecological momentary intervention (EMI), which is described as “treatments that are provided to people during their everyday lives (i.e., in real time) and in natural settings (i.e., real world) (by a mobile, electronic device)” (Heron & Smyth, 2010, p. 1). This method offers some important advantages. EMIs have high external validity, as the intervention takes place in real time in real life (Heron & Smyth, 2010). Considering the stressful daily work routine in nursing, it is particularly important to determine whether nurses actually succeed in putting an intervention method into practice. EMIs further have the benefit that the transfer of the intervention content to real life is greatly facilitated since the exercises take place directly in everyday life (Heron & Smyth, 2010). Resulting from EMI characteristics such as high privacy and flexibility (Heron & Smyth, 2010), an additional advantage of EMIs is the low threshold. EMIs offer a simple initial contact to an exercise method and may be suitable for generating interest in a topic such as mindfulness.

Another important characteristic of the study is the evaluation of intervention effects from a within-subject perspective. Many intervention studies follow a between-subject design and investigate differences between groups. We, on the contrary, address the question of whether the same nurses benefit from practicing mindfulness meditations compared to not practicing them by comparing differences between days with short meditations and control days during which nurses used their breaks as usual or started their after-work time as usual. Therefore, this design effectively controls between-subject differences.

### Promoting Recovery by Short Meditation Sessions

Two mechanisms of recovery can be distinguished. According to the effort-recovery model (Meijman & Mulder, 1998), recovery starts at the moment when the confrontation with demands ends, which has been called “a more passive mechanism” (Geurts & Sonnentag, 2006, p. 485). From this perspective, it is important for recovery during the working day that, for example, breaks are sufficiently long and free of job demands. On the other hand, the conservation of resources theory (Hobfoll, 2002) suggests that the activities pursued during the recovery period play an important role because some activities may be more effective in restoring resources than others. In the context of the “more active mechanism” of recovery (Geurts & Sonnentag, 2006, p. 485), the research question is to what extent certain activities are suitable to effectively restore stressed affective, cognitive, and physiological resources.

In field studies, various activities have been shown to be beneficial for recovery, for example, relaxation exercises (Riedl et al., 2023; Sianoja et al., 2018) and physical activity (Riedl et al., 2023; Sianoja et al., 2018). Mindfulness exercises may be a particularly promising activity, as they can function as a cognitive-emotional segmentation strategy (Althammer et al., 2021; Michel et al., 2014). In the meta-analysis by Karabinski et al. (2021), intervention programs including boundary management strategies and mindfulness activities were both positively associated with psychological detachment and showed a medium effect size. Mindfulness exercises foster the awareness of the present, work-free moment, for example, by feeling into the body, performing breathing techniques, or concentrating on sounds, which distracts the mind from work-related thoughts. Indeed, in the mixed samples of Michel et al. (2014) and Althammer et al. (2021), the participants of a 3-week online mindfulness training course showed an increase in daily psychological detachment over the study period. On this basis, we formulate the hypothesis that compared to control days, days with mindfulness meditations during breaks and after work are associated with higher break recovery and higher psychological detachment after work.

Successful recovery is highly important for well-being and performance (Bennett et al., 2018; Headrick et al., 2022; Steed et al., 2021). Among the different recovery conceptualizations (recovery activities, recovery experiences, and state of being recovered), the feeling of being recovered showed the strongest associations with the recovery outcome well-being, followed by psychological detachment (Steed et al., 2021). In the study by Riedl et al. (2023), break recovery was an important mediator of the associations between live-streaming activity and relaxation breaks and after-break mood and attention. In this study, taking into account the three mood dimensions of calmness (feeling calm and relaxed), valence (feeling comfortable and satisfied), and energetic arousal (feeling full of energy and wakeful; Wilhelm & Schoebi, 2007), we propose the following hypotheses: There are significant positive total associations between mindfulness meditations and after-break/after-sleep valence, calmness, and energetic arousal; the total association between meditations and after-break and after-sleep attention failures is negative; and break recovery and psychological detachment function as mediators of these associations.

## Methods

### Sample and Procedure

#### Study 1: Break Study

Data for the break study were collected over two periods at two German charitable associations of elderly care facilities. The first period took place in May 2021, and the second period took place in July and August 2022. In the instructions provided, nurses were asked to participate in the study for 12 shifts, if possible. The nurses were instructed to choose early or late shifts for their study participation, but not night shifts (because the work processes and breaks greatly differ here^1^). The app movisensXS was used for data collection and audio presentation (the audio files were also available for private download). According to two different versions of the instruction, half of the nurses were to start with a control day without meditation and the other half with an intervention day. On intervention days, nurses were invited to conduct one out of six short meditations during their break but additional to the regular break time, if possible (with the consent of the nursing management). Simple on-ear headphones were provided for carrying out the mindfulness exercises, which the nurses were allowed to keep after the study. Nurses were instructed to alternate intervention and control days on the remaining study days. In the second half of the working day, the nurses were asked to access the after-break questionnaire. As our focus is on within-subject associations, we aimed for a minimum sample size of *N*_2_ = 30 persons and *N*_1_ = 5 measurements (Arend & Schäfer, 2019) to have sufficient power to detect relationships of medium effect size. The evaluation was based on 38 elderly care nurses, who provided a total of 208 measurements. The individuals and measurement time points that were excluded are presented in the first section of the results, where we provide the details regarding feasibility and feedback.

#### Study 2: After-Work Study

Data for the after-work study were collected in a German general hospital in December 2022 and January 2023. The procedure was comparable with the break study but with the following differences: (a) Meditations were performed when arriving at home after work, (b) the after-sleep questionnaire was completed after sleep, and (c) every shift was allowed (night shifts, however, were rarely chosen). For the analysis, 193 measurements from 26 nurses were considered.

An approving institutional ethical vote has been received for both studies. Table 1 shows the demographic information for the two samples.

**Table 1.** Demographic Information for the Two Samples

	Break study (Study 1)	After-work study (Study 2)
Category	Frequency	
Sex		
Male	4	2
Female	33	23
Diverse	0	0
Age		
≤45 years	15	11
>45 years	22	14
Standard working time		
Fulltime	17	13
Part-time	20	12

*Note.* One person in each study did not complete the initial questionnaire.

### Short Audio-Guided Mindfulness Meditations

Audio mindfulness exercises were recorded by a female and a male yoga teacher, who both were very experienced in the context of work and health programs. Since experiencing control is an important recovery experience (Sonnentag & Fritz, 2007), we wanted to support this through the offer of choice, especially since matching the content of the meditation to individual preferences is important (Shapiro, 2021). To allow the nurses to explore personal preferences, both yoga teachers provided instructions for three mindfulness exercises that varied considerably from each other and included a body journey, a thought journey, a singing bowl meditation, and different types of breathing meditations that lasted between 7 and 8 minutes. All meditations were provided with accompanying relaxation music in the background. During the first study period of the break study, only three different meditations provided by the female yoga teacher were available.

### Measures

#### Self-Report Variables

For the break study (Study 1), the after-break survey asked nurses how long they had worked that day and how many minutes their break lasted. They were also asked to indicate whether the day was an intervention or control day and, on intervention days, whether they succeeded in performing the mindful break as planned and if not, why not (“I forgot,” “I did not have time for this,” “I had to interrupt the meditation”). Furthermore, they were asked to rate their break recovery. Recovery after breaks was assessed with a three-item short scale as described by Demerouti and colleagues (2012; e.g., “During the break I could recuperate”) using a seven-point Likert-type scale from *strongly disagree* (1) to *strongly agree* (7). The short scale of Wilhelm and Schoebi (2007) was used to assess momentary mood. This scale was constructed for the situational measurement of the three basic mood dimensions of valence, calmness, and energetic arousal and used two 7-point bipolar items for each dimension (e.g., “relaxed–tense”). The items were introduced by the question “How do you feel right now?”

For the after-work study (Study 2), the after-sleep questionnaire first asked nurses whether the proceeding study day was an intervention or a control day and, on intervention days, whether they succeeded in performing the meditation as planned and, if not, why (same answering options were provided as in the break study). Then, referring to the past after-work time, the nurses rated their psychological detachment using the four-item scale of the Recovery Experience Questionnaire by Sonnentag and Fritz (2007) in the after-work study. A sample item from this questionnaire was as follows: “On my closing day yesterday, I forgot about work.” To assess after-sleep mood, the short scale of Wilhelm and Schoebi (2007) was used again.

#### Attention Failures

The after-break and after-sleep questionnaires concluded with a short attention test. Attention failures were assessed by the Sustained Attention to Response Task (Robertson et al., 1997). This attention test is a go/no-go task in which the participants are instructed to react to every digit from 1 to 9 except 3 by touching the screen. While the original test lasts 4.3 minutes, we used a shortened version of approximately 1.5 minutes (Riedl et al., 2023). However, due to technical problems, 11 participants of the first wave of the break study received the original version instead of the shortened version. Therefore, we used the percentage of commission errors as the outcome variable. Test length did not affect this outcome variable (estimate = −0.02, *SE* = 0.08, *p* > .05).

### Data Analysis

The analyses were conducted with Mplus version 8.1.6. Multilevel path models were estimated to account for dependencies among the dependent variables. As the focus of both studies is on mediated relationships, it is important to account for the skewed sampling distribution of indirect effects (Preacher et al., 2010). Therefore, we used a Bayes approach, which flexibly handles abnormal distributions (Zyphur & Oswald, 2015) and provides 95% credibility intervals (CRIs) based on the posterior probability distribution of indirect effects. The posterior probability refers to the probability of the parameters after observing the data. CRIs represent the probable range of values for a certain effect, meaning that there is a probability of 95% that a certain effect ranges between the lower and upper limits of the interval (Zyphur & Oswald, 2015). We worked with the Mplus standard settings regarding the estimator Bayes (Markov chain Monte Carlo algorithm, noninformative priors; Muthén, 2010).

The mindfulness exercises were specified as a within-subject variable coded as 1 on intervention days and as 0 on control days. The other variables were modeled at both the within-subject and between-subject levels (Preacher et al., 2010). The intercepts were allowed to vary randomly between the participants, and the slopes were fixed (Preacher et al., 2010). The model fit was evaluated by posterior predictive checking, by which the probability of the observed data was compared with the probability of the data generated by posterior estimates of model parameters. The proportion of times that the observed data are more probable than the generated data is reflected by the posterior predictive *p* value, which should be near .50 because the observed data are just as probable as the generated data (Zyphur & Oswald, 2015). Furthermore, the confidence interval (CI) relating to the difference between the observed and generated data should include zero (Zyphur & Oswald, 2015).

In the tables, we present the StdY standardized estimates for associations of the binary variable mindfulness meditations and the StdXY standardized estimates for relationships between continuous variables. For the indirect and total associations between mindfulness meditations and the outcome variables, we provide partially standardized estimates of the indirect and total effects (MacKinnon, 2008), which are in the metric of standard deviation units of the dependent variables (Preacher & Kelley, 2011). Therefore, model constraints were set. In addition, based on the within-subject point-biserial correlations of the mindfulness intervention and the outcome variables, we provide *d* effect sizes (Ruscio, 2008).

## Results

### Feasibility of Conducting Break and After-Work Meditations and Participant Feedback

The descriptive statistics of the study variables are presented in Table 2.

**Table 2.** Descriptive Statistics of the Study Variables

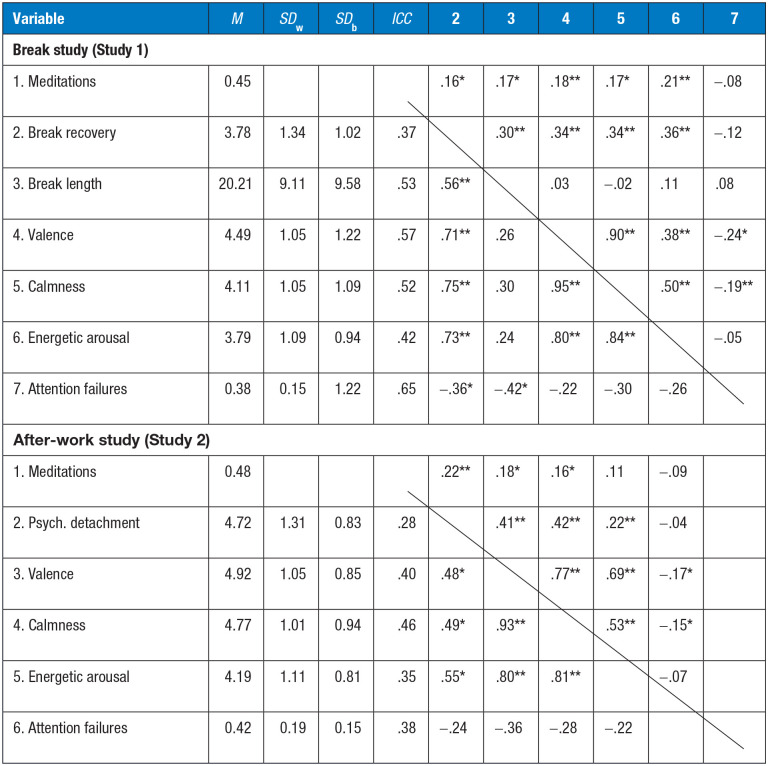

*Note. N*_2_ (persons) = 38 (break study)/26 (after-work study). *N*_1_ (self-report measures) = 208 (break study)/193 (after-work study). *N*_1_ (attention failures) = 185 (break study)/190 (after-work study) (reduced due to test interruptions). Above the diagonal, the within-person correlations are reported (except for the binary variable meditations, which were left in the metric 0 versus 1, these are based on person-mean centered variables), and below the diagonal, the between-person correlations based on aggregated data are shown. *SD*_w_ = within-subject standard deviation. *SD*_b_ = between-subject standard deviation. *ICC* = intraclass correlation.

* *p* < .05; **p < .01.

#### Study 1: Break Study

Eighty-one participants provided smartphone data; however, 36 nurses answered the initial questionnaire and/or attempted meditation but did not complete a questionnaire after the break. The inclusion criterion, defined as having at least two valid situational surveys (Nezlek, 2011), was fulfilled by 38 nurses who provided a total of 208 measurements. Questionnaires were classified as not valid if meditations were carried out on control days (20 measurements) or if the nurses indicated that the meditation was not performed as instructed (52 cases). In most cases (38), lack of time was the reason for the failure of the intervention. In another 11 cases, the break meditation was interrupted. In contrast, the intervention was seldom forgotten (in three cases).

Of the 208 valid after-break surveys, 114 referred to control days without a break intervention, and 94 referred to intervention days with successfully conducted meditations. On average, each nurse completed 5.5 break surveys. The after-break surveys were answered after an average of 6.7 hours of work. One hundred and seven measurements were completed after 6.5 working hours. According to the Occupational Health and Safety Act, a break of at least 30 minutes is required after 6 hours of work. During intervention days and after at least 6.5 working hours, only 18% of breaks were shorter than 30 minutes, while this was the case for almost every second break on control days (46%). Due to the instruction to add meditation to regular break time, we expected a positive association between intervention days and break length and considered it an alternative mediator of break recovery in the path model.

Thirty-one participants in the break study completed the feedback survey, in which the nurses were asked whether they liked the meditation exercises (liking), whether they could imagine continuing with the meditation exercises during the break (future practice), and whether they would like to see break exercises offered permanently on a voluntary basis (future offer). The mindfulness meditations were positively rated with an average above the midpoint of the scale, which is four (liking: *M* = 4.97, *SD* = 2.01; future practice: *M* = 4.42, *SD* = 2.13; future offer: *M* = 4.48, *SD* = 2.11). However, with a standard deviation above 2, there was also a high variance in the experience of the break meditations.

#### Study 2: After-Work Study

Originally, 41 nurses completed at least one questionnaire (initial questionnaire, meditation questionnaire or after-sleep questionnaire). However, the inclusion criterion of having completed at least two valid after-sleep questionnaires (Nezlek, 2011) was met by 26 nurses who provided 193 measurements (92 intervention days, 101 control days). Eighteen questionnaires were excluded because the nurses indicated that they had forgotten about performing meditation (six cases), had no time for mediation (four cases), or had to interrupt a meditation session (eight cases). In 24 cases, the after-work and after-sleep questionnaires were started simultaneously. Meditation performed on a control day occurred only once. On average, each nurse completed the after-sleep questionnaire 7.4 times. In the after-work study, 23 nurses provided clearly positive feedback (liking: *M* = 5.52, *SD* = 1.50; future practice: *M* = 5.26, *SD* = 1.74; future offer: *M* = 5.83, *SD* = 1.50).

### Study 1: Relationships of Short Mindfulness Meditations With Break Recovery and After-Break Well-Being and Attention

The model indicated an excellent fit (posterior predictive *p* value = .50; 95% CI [−40.64, 51.29]). Regarding the control variable break length, there was a significant positive association with mindful breaks, indicating longer breaks occurred on intervention days (unstandardized estimate = 3.95 minutes, CRI 95% [0.70, 6.59]). Break length and break recovery showed a significant positive correlation to moderate size (*r* = .28, CRI 95% [0.13, 0.39]). However, break length was not significantly associated with any of the outcome variables.

In agreement with the study hypothesis, mindfulness breaks were associated with higher break recovery than breaks as usual (see Table 3). Higher break recovery in turn significantly predicted higher after-break valence, calmness, and energetic arousal. Break recovery, however, was not significantly associated with a lower percentage of attention failures. As expected, short mindfulness meditations showed significant positive total associations with valence, calmness, and energetic arousal, which were partially mediated by break recovery (see Table 3). Thus, after participating in mindful breaks, the nurses felt more comfortable, calm, and energized than after control breaks without mindfulness exercises, which is due to the better break recovery. The effect sizes of the relationships between mindful breaks and break recovery (*d* = 0.33), valence (*d* = 0.37), calmness (*d* = 0.35), and energetic arousal (*d* = 0.43) are in the small to medium range (Cohen, 1988).

**Table 3.** Within-Person Path Coefficients of the Multilevel Path Model of the Break Study (Study 1)

	STDY/STDXY standardized estimates (CRIs) for the dependent variables					
	Break recovery	Break length	Valence	Calmness	Energetic arousal	Attention failures
*Independent variable*						
Meditations	**0.42 [0.13, 0.66]**	**0.42 [0.08, 0.71]**	**0.42 [0.10, 0.63]**	**0.39 [0.13, 0.59]**	**0.35 [0.05, 0.62]**	−0.22 [−0.56, 0.05]
*Mediators*						
Break recovery			**0.33 [0.18, 0.45]**	**0.35 [0.25, 0.48]**	**0.34 [0.20, 0.48]**	−0.16 [−0.29, 0.05]
Break length			−0.11 [−0.22, 0.06]	−0.15 [−0.26, 0.01]	−0.03 [−0.19, 0.07]	0.13 [−0.06, 0.31]
*Indirect association via break recovery*			**0.13 [0.04, 0.24]**	**0.13 [0.05, 0.26]**	**0.13 [0.04, 0.26]**	−0.06 [−0.15, 0.02]
*Total association*			**0.50 [0.19, 0.72]**	**0.45 [0.21, 0.67]**	**0.48 [0.20, 0.71]**	−0.22 [−0.58, 0.05]

*Note.* Credibility intervals (CRIs) that do not contain 0 are in bold. *N*_2_ (persons) = 38/*N*_1_ (self-report measures) = 208/*N*_1_ (attention failures) = 185 (reduced due to test interruptions). For the binary variable meditations, the StdY standardized estimates are reported, and for the continuous variables break recovery and break length, the StdXY standardized estimates are shown.

The indirect and total associations of mindful breaks and objectively measured attention narrowly missed the significance threshold. In the multilevel path model, correlations between the different dependent variables were controlled, and there were significant negative correlations between attention failures and the mood dimensions valence and calmness (see Table 2). Thus, we set a parsimonious model with attention failures as the only dependent variable and without considering the variable break length. With these constraints, there was a significant negative total association between mindful breaks and after-break attention failures (STDY standardized estimate = −0.28, CRI 95% [−0.58, −0.01]), which indicates that mindful breaks could indeed benefit after-break attention. However, the effect size of the association between mindful breaks and attention failures was only small (*d* = −0.16).

In terms of supplemental analyses, we investigated whether the effects of the mindfulness exercises were moderated by practice. Therefore, for each outcome variable, a multilevel model was constructed with the predictors of mindfulness exercise, number of measurements, and Mindfulness Exercise × Number of Measurements. However, practice effects were absent in any model.^2^

### Study 2: Relationships of Short Mindfulness Meditations With After-Work Detachment and After-Sleep Well-Being and Attention

With a posterior predictive *p* value of .47 and a CI for the difference between observed and replicated chi-square values of CI 95% [–12.97, 33.30], the mediation model for predicting after-sleep well-being and attention showed a good model fit. Supporting the hypothesis, the nurses reported higher after-work psychological detachment after intervention days than after control days (see Table 4). After-work psychological detachment in turn was positively associated with after-sleep valence, calmness, and energetic arousal. In line with the study hypotheses, the mindfulness intervention had significant positive total associations with after-sleep valence and calmness and psychological detachment played a mediating role in these associations. Thus, compared to days during which nurses carried out their after-work time as usual, the study participants reported better psychological detachment after days with mindfulness meditations, which benefits after-sleep mood in terms of valence and calmness.

**Table 4.** Within-Person Path Coefficients of the Multilevel Path Model of the After-Work Study (Study 2)

	STDY/STDXY standardized estimates (CRIs) for the dependent variables				
	Psychological detachment	Valence	Calmness	Energetic arousal	Attention failures
*Independent variable*					
Meditations	**0.44 [0.18, 0.74]**	0.19 [−0.05, 0.46]	0.13[−0.14, 0.42]	0.13 [−0.12, 0.41]	−0.16 [−0.52, 0.12]
*Mediators*					
Psychological detachment		**0.39 [0.26, 0.51]**	**0.41 [0.28, 0.52]**	**0.20 [0.05, 0.35]**	−0.02 [−0.19, 0.15]
*Indirect association via psychological detachment*		**0.17 [0.07, 0.31]**	**0.18 [0.07, 0.33]**	**0.09 [0.02, 0.20]**	−0.01 [−0.10, 0.08]
*Total association*		**0.36 [0.08, 0.67]**	**0.31 [0.03, 0.62]**	0.22 [−0.02, 0.50]	−0.17 [−0.51, 0.12]

*Note.* Credibility intervals (CRIs) that do not contain 0 are in bold. *N*_2_ (persons) = 26/*N*_1_ (self-report measures) = 193/*N*_1_ (attention failures) = 190 (reduced due to test interruptions). For the binary variable meditations, the StdY standardized estimates are reported, and for the continuous variable psychological detachment, the StdXY standardized estimates are shown.

In addition, there was a significant positive indirect relationship between mindfulness meditations and after-sleep energetic arousal. The total association between mindfulness meditations and after-sleep energetic arousal, however, was not significant. Indirect or total associations between the mindfulness intervention and objectively measured attention were absent, as were the moderating effects of practice.^3^ The strongest effect size was found for psychological detachment (*d* = 0.45). However, for energetic arousal (*d* = 0.22) and attention (*d* = −0.18), the associations with the intervention were small, and the relationships for valence (*d* = 0.36) and calmness (*d* = 0.32) were small to medium.

## Discussion

This study evaluated the feasibility and effectiveness of short mindfulness meditations in nursing. One study investigated the associations of short break meditations in geriatric nursing, while in the other study, nurses working in a general hospital performed meditations after shift. In both studies, the short mindfulness meditations were associated with a better recovery experience (related to break recovery and psychological detachment, respectively). Mediated by the recovery advantage, break- and after-work meditations were associated with higher after-break and after-sleep valence and calmness. While in the break study, the short mindfulness mediations were additionally associated with higher after-break energetic arousal, and for after-sleep energetic arousal, only the indirect relationship via psychological detachment was significantly positive. It is plausible that the lack of a total association between after-work meditation and after-sleep energetic arousal may be attributed to the higher time difference between the meditation and the after-meditation questionnaire. For after-sleep wakefulness and energy, variables such as the type of previous shift and sleep quality are important (Niu et al., 2011). The same reasons may explain the lacking indirect and total associations regarding the objectively measured attention performance in the after-work study.

In the break study (Study 1), the negative relationship between mindfulness meditations and attention failures was not significant in the multivariate path model. In the path model, correlations between the different dependent variables were controlled, and valence/calmness and attention failures showed a negative relationship, indicating that positive and relaxed after-break mood is associated with fewer attention failures. In a simpler model without considering the variable break length and with attention failures as the only dependent variable, the total association between mindful breaks and after-break attention failures was significantly negative. Thus, short mindfulness meditations during breaks may be beneficial for after-break attention, but not independently of the self-reported mood variables. Furthermore, we assume that the measurement of attention performance is considerably susceptible to disruptions in everyday nursing care. This may explain why the relationships of our study variables with attention failures were rather weak. In this case, a higher statistical power would be necessary to detect robust relationships, and influencing factors such as daily demands should be controlled. However, in light of the importance of attention for workplace safety and performance (Allan et al., 2014; Park & Kim, 2013), we see great potential in ambulatory attention measurement for stress (Allan et al., 2014) and recovery research (Riedl et al., 2023).

Overall, this study showed that short mindfulness meditations are helpful in nursing for promoting break recovery and after-work recovery. On the other hand, the break study (study 1) clearly shows that, in view of the highly problematic status quo regarding break times in geriatric care, the feasibility of break meditations in nursing is currently limited. Quite a few nurses (eight individuals) did not manage to take a single successful mindful break during the study period, although they regularly participated in the study and provided several (control) measurements. Thus, the very promising results regarding mindful meditations during breaks must be viewed with caution because the effects are limited to those nurses who succeeded in integrating the meditations into their stressful daily work routine.

The effect sizes of the mindfulness exercises in both studies ranged from small to medium (Cohen, 1988). Despite major differences in the study designs, the effect size in the after-work study relating to detachment (*d* = 0.45) was very close to the average effect size of detachment interventions including mindfulness (*d* = 0.46) in the meta-analysis by Karabinski et al. (2021). The correlations between the intervention and the outcome variables observed in the break study (Study 1) were comparable to those in other break studies including relaxation breaks conducted in other occupational settings (Riedl et al., 2023; Sianoja et al., 2018).

Other studies (e.g., Hülsheger et al., 2015) found significant interactions between the mindfulness intervention and time. In this study, the number of measurements did not moderate the relationships of the meditation exercises. The meditations have been created specifically so that they can be performed successfully for the first time without any previous experience. This objective could explain why our studies show no practice effects.

### Implications for Occupational Health Practice

Overall, it can be concluded that, first, all nurses should generally have the opportunity to take a break of an appropriate duration without interruptions and disturbances before designing break content to promote recovery. Without a preceding structural break intervention, a break intervention would only be of limited use for the nursing staff. Wendsche et al. (2022), for example, recommend daily break planning and break areas, but workplace cultures and individual beliefs regarding self-care in nursing also represent crucial considerations (Poulsen et al., 2015). Break length was only moderately positively correlated with break recovery and did not predict any of the outcome variables beyond break recovery, while break recovery was an important predictor for the three after-break mood dimensions. Therefore, after ensuring a sufficiently long break for every nurse, an offer of short mindfulness meditations should be established and ideally, as in this study, be advertised in addition to the regular break duration. Regular break time is needed to satisfy personal needs, and nurses usually spend their breaks with colleagues (Wendsche et al., 2022), which should not be interfered with.

A very interesting alternative involves combining short mindfulness meditations with virtual reality, as described by Pascual and colleagues (2023). Their study, which was conducted in an emergency department, demonstrated that virtual reality meditations were more effective regarding heart rate variability than mobile meditations alone, while both types were associated with decreased self-reported anxiety. The authors concluded that, particularly regarding routine use, virtual reality meditations may be superior to audio-only meditations. Regarding expansion options, physiotherapeutic content could be included, for example, to address physical issues related to the back.

Another way to promote the effectiveness of mindfulness exercises during breaks could be to optimize the spatial conditions and to provide quiet and comfortable break rooms. In our study, the nurses were instructed to find a quiet place with a seat for the meditation breaks. Given that the break rooms of the care facilities were not ideal, the nursing staff were provided with simple on-ear headphones for the meditation sessions.

In the after-work study (Study 2), no nurses who participated regularly failed to implement the intervention. As positive associations with psychological detachment and after-sleep valence and calmness were found and harmful effects were absent, it can be recommended to offer and advertise mindfulness exercises during after-work time. An important area for future research is to investigate the effects of longer-term usage of such offerings in the field of nursing. Even though some nurses indicated in the evaluation that they wanted to continue the meditations, the extent to which they truly made use of the meditations that were made available on a permanent basis remains unclear. Recovery from job-related strain is crucial for long-term mental and physical well-being and performance, while high-stress working conditions constitute an important recovery hindrance (Sonnentag, 2018). Thus, it is economically advantageous for employers to invest in the recovery of their employees. Ideally, recovery programs take place during working hours, but if this is not possible, then approaches should be sought to enable this investment of free time by nurses. Due to serious staff shortages in the care sector (Bundesanstalt für Arbeitsschutz und Arbeitsmedizin, 2020), additional short breaks or working time credits for after-work activities are difficult to implement. This results in a double paradox: What necessitates recovery not only hampers the recovery process (Sonnentag, 2018) but also hinders measures to promote recovery. However, perhaps monetary incentives could be created to address this, possibly in cooperation with health insurance companies.

**Applying Research to Occupational Health Practice**

This study evaluated whether short mindfulness meditations performed during breaks or after work improve recovery in nursing. Compared to control days during which nurses used their breaks as usual or started their after-work time as usual, short meditations were positively related to break recovery and psychological detachment after work. Successful recovery, in turn, predicted a more positive mood.

While offering mindfulness exercises during after-work time can be recommended, the feasibility of break meditations is currently limited due to the problematic status quo regarding break times in nursing. Before designing break content to enhance break recovery—which can be explicitly recommended in light of the study results—structural changes are necessary to ensure consistent implementation of breaks of appropriate duration.

## Notes

1. The nurses were not instructed to vary the shift type during their study participation. In both studies, many nurses did not provide data for different shift types. Because of the within-subject focus of the analyses, we do not consider shift type as a variable.

2. STDY standardized estimates of the interaction between the mindfulness intervention and the number of measurements: Recovery after break: estimate = −0.02, CRI 95% [−0.11, 0.06]; valence: estimate = −0.08, CRI 95% [−0.17, 0.004], calmness: estimate = −0.07, CRI 95% [0.16, 0.01], energetic arousal: estimate = −0.06, CRI 95% [−0.15, 0.03], attention failures: estimate = 0.09, CRI 95% [−0.06, 0.19].

3. STDY standardized estimates of the interaction between the mindfulness intervention and the number of measurements: Detachment: estimate = 0.04, CRI 95% [−0.03, 0.12]; valence: estimate = 0.04, CRI 95% [−0.04, 0.11]; calmness: estimate = 0.02, CRI 95% [−0.05, 0.10]; energetic arousal: estimate = −0.01, CRI 95% [−0.08, 0.07]; attention failures: estimate = −0.04, CRI 95% [−0.11, 0.04].
